# Regulation of gene expression in human tendinopathy

**DOI:** 10.1186/1471-2474-12-86

**Published:** 2011-05-03

**Authors:** Scott A Jelinsky, Scott A Rodeo, Jian Li, Lawrence V Gulotta, Joanne M Archambault, Howard J Seeherman

**Affiliations:** 1 Tissue Repair, Pfizer Research, 200 Cambridge Park Drive, Cambridge, MA 02140 USA; 2 Section of Shoulder & Sports Medicine, Laboratory for Soft Tissue Research, Hospital for Special Surgery, 535 East 70th Street, New York City, NY 10021, USA

## Abstract

**Background:**

Chronic tendon injuries, also known as tendinopathies, are common among professional and recreational athletes. These injuries result in a significant amount of morbidity and health care expenditure, yet little is known about the molecular mechanisms leading to tendinopathy.

**Methods:**

We have used histological evaluation and molecular profiling to determine gene expression changes in 23 human patients undergoing surgical procedures for the treatment of chronic tendinopathy.

**Results:**

Diseased tendons exhibit altered extracellular matrix, fiber disorientation, increased cellular content and vasculature, and the absence of inflammatory cells. Global gene expression profiling identified 983 transcripts with significantly different expression patterns in the diseased tendons. Global pathway analysis further suggested altered expression of extracellular matrix proteins and the lack of an appreciable inflammatory response.

**Conclusions:**

Identification of the pathways and genes that are differentially regulated in tendinopathy samples will contribute to our understanding of the disease and the development of novel therapeutics.

## Background

Chronic injuries to the Achilles, patellar, extensor carpi radialis brevis, and supraspinatus tendons remain a common problem for both elite and recreational athletes, as well as for individuals engaging in repetitive activities. These overuse type injuries account for 30-50% of all sports injuries and result in a significant amount of morbidity and health care expenditure [1].

Histologic studies have shown that the primary pathology is not inflammation as implied by the commonly used term "tendonitis." Instead, samples of diseased tendons show collagen degeneration, fiber disorientation, mucoid ground substance, hypercellularity, vascular ingrowth, and relative absence of inflammatory cells under light microscopy [2-4]. Tendinopathy (or tendinosis) is now the term most commonly used to describe the clinical entity and histologic findings. Interestingly, these findings are common to all tendinopathies, suggesting a similar etiology and pathophysiology.

The etiology of tendinopathy remains unclear, but most believe that a combination of extrinsic and intrinsic factors is responsible. The extrinsic theory suggests that direct mechanical contact leads to tendon fiber micro-damage and subsequent injury of the tendon that eventually results in weakness and pain. An example is impingement of the acromion on the supraspinatus tendon, which serves as the rationale behind acromioplasty surgery [5]. The intrinsic theory suggests that the tendon itself becomes inherently degenerative, probably as a result of microscopic fiber failure leading to accumulation of damage due to inability of the tendon to self-repair. Local ischemia may also be a contributory factor. Studies on the supraspinatus tendon have shown that its mid-portion is relatively hypovascular [6]. This lack of perfusion may result in the formation of oxygen free radicals or other molecules that initiate the pathological process.

Several observations have been made about the molecular mediators of tendinopathy. Tenocyte apoptosis or "programmed cell death" has been shown to occur at an increased frequency in tendinopathy specimens [7]. Free radicals as well as cyclic loading may induce the activation of molecules that lead to apoptosis [8,9]. In addition, animal studies have shown that various cytokines and matrix metalloproteinases (MMPs) may be disproportionately expressed in tendinopathy specimens. The application of cyclic strain has been shown to increase the production of prostaglandin E2 (PGE2), interleukin-6 (IL6), and IL1β [10,11]. IL1β in turn increases the production of MMP1, MMP3, and PGE2 [12]. Alfredson et al. studied samples from patients with Achilles tendinopathy and found downregulation of *MMP3 *mRNA and upregulation of *MMP2 *and vascular endothelial growth factor compared with control samples [13]. Riley et al. reported decreased *MMP3 *and *MMP2 *mRNA activity, with an increase in *MMP14 *[14]. These studies show that an imbalance in cytokines and MMPs exists in diseased tendons and probably contributes to the pathophysiology; however, inconsistencies in the expression of specific molecules in various studies indicate that more research needs to be done.

Currently, the only non-surgical therapies available to patients who suffer from chronic tendinopathies are physical therapy, activity modification, non-steroidal anti-inflammatory medications (NSAIDs), and steroid or platelet-rich plasma injections. These therapies offer unpredictable results, and in the case of steroids, can lead to serious side effects and more rapid degeneration of the tendon. By understanding the molecular mediators that lead to tendinopathy, novel therapeutic targets could potentially be identified for drug development. This will result in more effective treatments while minimizing side effects.

Microarray analysis has become a powerful tool in drug development. Microarrays allow researchers to screen samples of tissue for the expression of thousands of genes encoded in the human genome. This "shotgun" approach can identify which genes are active in a given sample by quantifying the production of specific mRNAs. Identification of the products of these mRNAs and their functions can serve to direct future research and development.

The purpose of this study was to investigate whether there is selective regulation of certain cytokines, matrix metalloproteinases, and protein kinases in human samples of tendinopathy compared with normal tendons, as evidenced by microarray analysis. Specifically, we were interested in determining which specific cytokines and degenerative enzymes are upregulated, and which are downregulated, in diseased samples compared with normal control tendons from the same patients. This information may contribute to the development of drugs that can selectively modulate the disease process.

## Methods

### Subjects

All protocols were approved by the Institutional Review Board at the Hospital for Special Surgery and informed consent was obtained from each participant. A prospective study was initiated to collect tissue from patients undergoing surgery as standard of care for tendinopathy. Biopsies (~3 mm^3^) of diseased tendons and a section of grossly normal appearing tendon were collected from 35 patients. Written informed consent was obtained from all patients prior to any study-related procedure. Information on patient demographics, diagnosis, prior treatments, current procedures, and operative findings was collected and is presented in Table 1. Patients with a diagnosis of inflammatory arthritis (such as rheumatoid arthritis or lupus) or those who had prior surgery on the involved tendon were excluded. Patients who received prior corticosteroid injection were included and this information was recorded.

**Table 1:** Demographics and patient clinical diagnosis

ID	Age	Sex	Diagnosis	Duration of Sx (months)	Num of Inject*	Pathology Sample	Control Sample
5	45	M	Distal Biceps Rupture	0.3		Distal Biceps	Brachialis
9	48	M	ECRB tear	12		ECRB	EDC
10	41	M	Patella Tendon Rupture	0.1		Patella	Quad tendon
12	62	M	Flexor/Pronator	18	1	flexor/pronator	Distal Flexor/Pronator
13	62	F	RTC tear	48	2	Supraspinatus	Subscap
15	32	M	Patella Tendon Tear	3		Patella tendon	Nl Patellar Tendon
16	61	F	RTC tear	24	2	Supraspinatus	Subscap
17	59	M	RTC tear	6	1	Supraspinatus	Subscap
19	45	M	Flexor/Pronator	18	3	flexor/pronator	Distal Flexor/Pronator
20	63	F	RTC tear	9	1	Supraspinatus	Subscap
21	65	F	RTC tear	3	1	Supraspinatus	Biceps
23	64	F	RTC tear	8	1	Supraspinatus	Subscap
24	50	M	RTC tear	18	1	Supraspinatus	Biceps
26	55	M	ECRB tear	4		ECRB	Distal ECRB
27	50	M	RTC tear	4		Supraspinatus	Normal RTC
28	41	M	Supra tear (PT), OA, Bankart	>24		Supraspinatus	Biceps Tendon
29	46	M	RTC tear, Adhesive Capsulitis	4		Supraspinatus	Biceps Tendon
30	52	M	ECRB tear	12		ECRB	ECRL
31	66	M	RTC tear	6		Supraspinatus	Subscap
32	46	F	RTC tear	2	1	Supraspinatus	Teres minor
33	44	M	RTC tear	6	1	Supraspinatus	Subscap
34	59	F	RTC tear	1	3	Supraspinatus	Biceps
35	49	F	RTC tear	24		Supraspinatus	Subscap

* Number of corticosteroids injections

ECRB Extensor Carpi Radialis Brevis

RTC Rotator Cuff

OA Osteoarthitis

EDC extensor digitorum comunis

### RNA Preparation and Hybridization

RNA extraction and isolation were performed as previously described [15]. Fifty nanograms of RNA was labeled using the WT-Ovation FFPE System (Nugen, San Carlos, CA). Labeled cDNA was hybridized to GeneChip^® ^Human Genome U133 2.0 arrays according to the manufacturer's protocol (Affymetrix, Santa Clara, CA). For each array, all probe sets were normalized to a mean signal intensity value of 100. The default GeneChip Operating Software statistical values were used for all analyses. The raw microarray data has been deposited in the Gene Expression Omnibus (GEO) database http://www.ncbi.nlm.nih.gov/projects/geo/ as accession number GSE26051.

### Identification of Differentially Expressed Genes

Expression values for all probe sets were subjected to locally weighted scatterplot smoothing (LOWESS) transformation. Correlation analysis and hierarchical clustering verified that all samples had similar patterns of expression. Only transcripts that were called as present in 65% of the samples and expressed greater than 35 signal units in either the normal or diseased samples were used for further analysis. Expression values were log2 transformed and transcripts were considered differentially regulated if the p-value based on a paired t-test analysis was < 0.01 and the average fold change was greater than 1.5. Extensive clinical information was collected for each patient. Analysis of variance models were constructed to determine whether any differences in expression could be explained by differences in histological disease score, disease duration, gender, steroid use, or NSAID use. Values presented are expressed as mean ± standard error of the mean. Differences were considered statistically significant for *p *< 0.01.

### Identification of Significantly Regulated Gene Sets

Significantly regulated biological pathways were identified using a modified version of the sigPathway algorithm [16] incorporating a modified normalization routine and using gene sets collections C1 to C5 defined by the Molecular Signatures Database (MSigDB) [17]. A gene set was considered significant when q1 ≤0.05 and q2 ≤0.05, where q1 or q2 are the permutation-based false-discovery rates for the Q1 or Q2 hypotheses (see [18] for an explanation of Q1 and Q2). These cut-offs were met by1761 gene sets, which can be considered as differentially expressed.

### Histology

Tissue samples were frozen and sectioned for histological analysis. Sections were processed using standard procedures, including Alcian blue (AB) staining. Sections were scored on four categories: intratendinous cellular proliferation, intratendinous vascular proliferation, intratendinous glycosaminoglycan (GAG) accumulation, and intratendinous fiber disorientation. Each category was scored based on a 5-point scale, from 0 for normal, 3 for moderate, and 5 for severe, by two independent histologists.

## Results

### Analysis of Tendinopathy Tissue Samples

**Figure 1:**
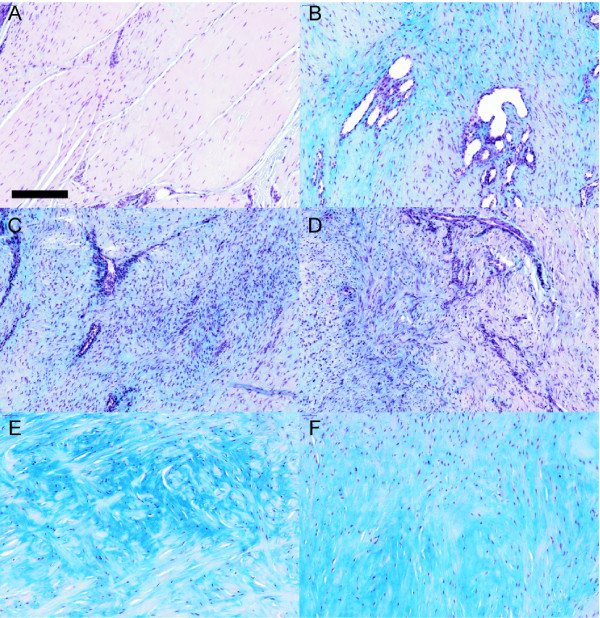
**Histological evaluation of human tendinopathy**. Representative diseased tendons were stained with Alcian blue to visualize the proteoglycans in patient 12 (A), patient 10 (B), patient 15 (C), patient 24 (D), patient 25 (E), and patient 32 (F). Only a few bundles from patient 12 appear normal. The remaining sections show highly disorganized tissues with increased cell proliferation, increased vasculature, and/or increased GAG content. Scale bar is 200 μM.

Diseased and normal tendons were collected from 35 patients with rotator cuff tendinopathy, lateral epicondylitis (tennis elbow), patellar tendinopathy, or chronic Achilles tendinopathy who were undergoing surgery as standard of care treatment for tendinopathy. Tendinopathy was confirmed both macroscopically and microscopically. The diseased tendon appeared dull, yellow, and soft compared with normal tissue, which is shiny, white, and firm. Normal tendon tissue had relatively few cells, contained little vasculature, and had collagen fibers aligned in the direction of force. All diseased tendons had a similar loss of normal fibrillar structure and changes in cell orientation, which are histological characteristics of tendinopathy [2,19,20]. All diseased tissue had an increased number of fibroblasts, increased vasculature, and increased glycosaminoglycan staining (GAG content) although the degree of increase in each of the parameters was variable between patients (Figure 1, Table 2). There was little evidence of any inflammatory cells in either the tendon or the surrounding paratenon.

**Table 2:** Intratendinous tendinopathy score^a^

Patient^b^	Cellular Proliferation	Vascular Proliferation	GAG Accumulation	Fiber Disorientation	Total Score
5	3.5	1.5	1.5	5	11.5
9	5	2	5	5	17
10	4.5	5	5	5	19.5
12	4	5	1	5	15
13	5	1	3.5	5	14.5
15	5	5	5	4.5	19.5
16	3.5	2.5	2	5	13
17	5	4.5	3	5	17.5
19	4.5	5	3	5	17.5
20	5	3	3	5	16
21	5	1	2	5	13
23	4.5	3	4	5	16.5
24	5	5	3	5	18
26	5	3	5	5	18
27	4.5	3	1	5	13.5
28	5	1.5	5	5	16.5
29	5	5	5	5	20
30	4	4.5	3	4.5	16
31	4.5	1.5	4	5	15
32	4.5	1.5	5	5	16
33	5	0.5	4	5	14.5
34	5	0.5	3	5	13.5
35	5	5	3	5	18

^a ^Average of two independent histologist evaluation

^b ^Normal tendon were scored 0 in all fields

### Analysis of Gene Expression

**Figure 2:**
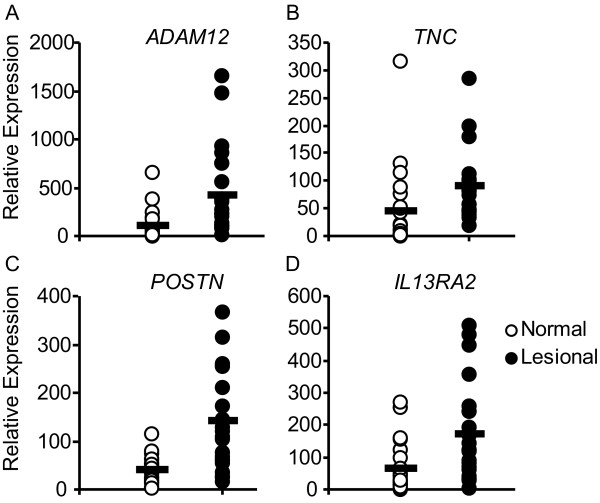
**Expression levels of genes that are differentially regulated in tendinopathy**. Messenger RNA expression levels for (A) a disintegrin and metalloproteinase domain 12 (*ADAM12*), (B) tenascin C (*TNC*), (C) periostin (*POSTN*), (D) interleukin 13 receptor alpha 2 (*IL13RA2*) in tendons from patients with tendinopathy. Values represent the relative expression values as determined by microarray analysis.

Global transcriptional analysis using Affymetrix genome-wide U133 2.0 Plus arrays identified 20055 transcripts expressed in 23 pairs of normal and diseased tendons that passed quality control filters. Of these, 983 transcripts were differentially regulated based on a paired t-test (p < 0.01, fold change ≥1.5. Among the top regulated genes were a disintegrin and metalloprotease 12 (*ADAM12*), tenascin C (*TNC*), periostin (*POSTN*), and interleukin 13 receptor alpha 2 (*IL13RA2*; Figure 2, Table 3). An analysis of covariance (ANCOVA) showed little relationship between the age of the patient and the level of gene expression for most transcripts. Furthermore, ANCOVA analysis suggested that < 1.2% of the transcripts showed any relationship between gene expression and histological disease score, disease duration, steroid use, NSAID use, gender, or duration of symptoms.

**Table 3:** Top regulated transcripts in human tendinopathy as determined by microarray analysis

Gene Symbol	Description	p-value	q-value	Fold Change
*ADAM12*	A disintegrin and metalloproteinase domain 12	1.57E-05	2.09E-04	5.6
*AP3M1*	Adaptor-related protein complex 3, mu 1 subunit	1.24E-03	1.91E-03	1.7
*ARL7*	ADP-ribosylation factor-like 7	1.43E-03	2.11E-03	4.0
*ARPC5*	Actin related protein 2/3 complex, subunit 5	3.63E-05	2.10E-04	1.8
*ASXL1*	Additional sex combs like 1 (Drosophila)	1.11E-03	1.78E-03	1.8
*ATP6V1A*	ATPase, H+ transporting, lysosomal 70 kDa	4.18E-05	2.10E-04	2.5
*BACE2*	Beta-site APP-cleaving enzyme 2	3.17E-06	8.01E-05	1.9
*BNC2*	Basonuclin 2	1.08E-04	3.15E-04	2.9
*CA12*	Carbonic anhydrase XII	3.57E-05	2.10E-04	2.8
*CTHRC1*	Collagen triple helix repeat containing 1	5.20E-04	1.10E-03	4.4
*DDEF1*	Development and differentiation enhancing factor	2.83E-05	2.10E-04	2.1
*DOCK10*	Dedicator of cytokinesis 10	7.91E-03	8.11E-03	3.3
*EDIL3*	EGF-like repeats and discoidin I-like domains 3	4.80E-03	5.34E-03	2.9
*FOXP1*	Forkhead box P1	4.67E-05	2.10E-04	1.7
*GPR161*	G protein-coupled receptor 161	2.76E-04	6.89E-04	1.9
*IGFBP3*	Insulin-like growth factor binding protein 3	7.29E-03	7.67E-03	3.5
*IL13RA2*	Interleukin 13 receptor, alpha 2	2.80E-03	3.50E-03	3.5
*ILK*	Integrin-linked kinase	1.00E-04	3.15E-04	1.6
*IQGAP1*	IQ motif containing GTPase activating protein 1	4.73E-05	2.10E-04	1.5
*ITGB1*	Integrin, beta 1	3.52E-04	8.29E-04	1.7
*ITPKB*	Inositol 1,4,5-trisphosphate 3-kinase B	7.37E-04	1.34E-03	2.5
*JAG1*	Jagged 1 (Alagille syndrome)	1.90E-03	2.52E-03	3.1
*KCNE4*	Potassium voltage-gated channel 4	8.19E-03	8.19E-03	3.4
*LAMA4*	Laminin, alpha 4	6.73E-04	1.31E-03	3.4
*LRRC15*	Leucine rich repeat containing 15	3.50E-03	4.24E-03	3.2
*LRRC17*	Leucine rich repeat containing 17	1.74E-03	2.40E-03	2.8
*MARCKS*	Myristoylated alanine-rich kinase C substrate	3.93E-03	4.50E-03	2.8
*NOTCH3*	Notch homolog 3 (Drosophila)	1.65E-03	2.36E-03	3.6
*PDIR*	protein disulfide isomerase-related	4.86E-04	1.08E-03	2.1
*POSTN*	Periostin, osteoblast specific factor	1.10E-04	3.15E-04	3.5
*RAP1GDS1*	RAP1, GTP-GDP dissociation stimulator 1	1.01E-03	1.68E-03	2.1
*S100A10*	S100 calcium binding protein A10	1.95E-03	2.52E-03	2.8
*SIN3A*	SIN3 homolog A, transcription regulator (yeast)	9.51E-05	3.15E-04	1.7
*SLC2A13*	Solute carrier family 2 13	5.41E-03	5.85E-03	2.8
*TAOK1*	TAO kinase 1	1.51E-04	4.03E-04	1.7
*TJP1*	Tight junction protein 1 (zona occludens 1)	6.87E-04	1.31E-03	1.6
*TNC*	Tenascin C (hexabrachion)	9.20E-05	3.15E-04	5.3
*UBE2E3*	Ubiquitin-conjugating enzyme E2E 3	8.72E-04	1.52E-03	3.7
*WDR1*	WD repeat domain 1	4.01E-06	8.01E-05	1.6
*WISP1*	WNT1 inducible signaling pathway protein 1	3.93E-03	4.50E-03	3.4

Global pathway analysis, based on a modified sigPathway algorithm [16], identified increased expression of genes involved in the extracellular matrix, including genes involved in focal adhesion, integrin signaling, and collagen synthesis. In addition, expression of genes involved in cell cycle progression, TGFβ signaling, and NFκB signaling was increased (Table 4). This global pathway analysis did not suggest that cytokines as a whole were differentially regulated. However, of the 426 transcripts annotated by Gene Ontology as cytokines, cytokine signaling molecules, or related to cytokines, six were differentially regulated in diseased tendons, including chemokine-like factor super family 4 (*CMTM4*), interleukin 13 receptor alpha 2 (*IL13RA2*), interleukin 17D (*IL17D*), and interleukin 4 receptor (*IL4R*) (Table 5).

**Table 4:** Genesets regulated in human tendinopathy as determined by sigpathway

	NTk*
Up regulated	
HSA04110_CELL_CYCLE	6.52
RNA_PROCESSING	6.44
SPINDLE	6.25
HSA04510_FOCAL_ADHESION	6.10
TGFBETA_LATE_UP	6.02
CELL_CYCLE_KEGG	6.00
CELL_CYCLE	5.94
ST_INTEGRIN_SIGNALING_PATHWAY	5.40
COLLAGEN	5.32
RUIZ_TENASCIN_TARGETS	5.24
I_KAPPAB_KINASE_NF_KAPPAB_CASCADE	5.23
MITOSIS	5.03
INOS_ALL_UP	4.89
Down Regulated	
ION_TRANSMEMBRANE_TRANSPORTER_ACTIVITY	-4.99
G_PROTEIN_COUPLED_RECEPTOR_ACTIVITY	-5.01
ELECTRON_TRANSPORT_CHAIN	-5.21
MOOTHA_VOXPHOS	-6.01
ION_CHANNEL_ACTIVITY	-6.20
SUBSTRATE_SPECIFIC_CHANNEL_ACTIVITY	-6.27

* As defined in Tian et. al [18]

**Table 5:** Chemokines regulated in human tendinopathy

Gene Symbol	Description	p-value	q-value	Fold Change
*BMP1*	Bone morphogenetic protein 1	0.000	0.005	1.69
*BMP8B*	Bone morphogenetic protein 8b	0.002	0.011	1.79
*CCL2*	Chemokine (C-C motif) ligand 2	0.005	0.014	1.74
*CMTM4*	Chemokine-like factor super family 4	0.008	0.017	1.98
*CMTM6*	Chemokine-like factor super family 6	0.014	0.023	1.37
*IFNGR1*	Interferon gamma receptor 1	0.027	0.034	1.42
*IL13RA2*	Interleukin 13 receptor, alpha 2	0.003	0.011	3.48
*IL17D*	Interleukin 17D	0.008	0.017	0.54
*IL6R*	Interleukin 6 receptor	0.026	0.034	0.73
*LEPR*	Leptin receptor	0.028	0.034	0.49
*OSMR*	Oncostatin M receptor	0.038	0.043	1.81
*SP110*	SP110 nuclear body protein	0.010	0.019	1.51
*STAT2*	Signal transducer and activator of transcription 2	0.051	0.054	1.4
*STAT3*	Signal transducer and activator of transcription 3	0.004	0.013	1.87
*TXLNA*	Taxilin	0.001	0.006	1.39

Consistent with a role in increased matrix turnover, tendonopathy tissue exhibited increased expression of genes encoding MMPs (Table 6) and genes encoding collagens (Table 7). The majority of collagens interrogated showed a modest 2-fold increase in expression in diseased tendons. Diseased tendons also showed increased expression of several enzymes that mediate collagen breakdown, including *MMP2, MMP9, MMP14, MMP13*, and *MMP19*. While many MMP genes were induced in diseased tendons, not all transcripts encoding MMPs were upregulated; in fact, *MMP3 *and *MMP24 *were significantly downregulated in diseased tissue. Of the four known tissue inhibitors of metalloproteases (TIMPs), only expression of *TIMP1 *was increased. No ADAMTS (a disintegrin and metalloprotease with thrombospondin motifs) family members were differentially regulated based on our cut-offs.

**Table 6:** Change in matrix metallopeptidase gene expression in human tendinopathy

Gene Symbol	Description		p-value	q-value	Fold Change
Regulated					
*MMP2*	Matrix metallopeptidase 2	Expressed	7.42E-03	4.82E-02	1.8
*MMP3*	Matrix metallopeptidase 3	Expressed	5.50E-02	2.38E-01	0.3
*MMP14*	Matrix metallopeptidase 14	Expressed	1.79E-03	1.66E-02	1.9
*MMP19*	Matrix metallopeptidase 19	Expressed	1.91E-03	1.66E-02	3.4
Not Regulated					
*MMP28*	Matrix metallopeptidase 28	Expressed	0.09	0.30	1.4
*TIMP1*	TIMP metallopeptidase inhibitor 1	Expressed	0.10	0.30	1.6
*TIMP2*	TIMP metallopeptidase inhibitor 2	Expressed	0.37	0.50	1.1
*TIMP3*	TIMP metallopeptidase inhibitor 3	Expressed	0.26	0.49	0.9
*TIMP4*	TIMP metallopeptidase inhibitor 4	Expressed	0.71	0.80	0.9
Expressed below limited of detection					
*MMP1*	Matrix metallopeptidase 1	-	0.96	0.96	1.0
*MMP7*	Matrix metallopeptidase 7	-	0.34	0.49	0.8
*MMP8*	Matrix metallopeptidase 8	-	0.81	0.84	0.8
*MMP9*	Matrix metallopeptidase 9	-	0.00	0.02	3.2
*MMP10*	Matrix metallopeptidase 10	-	0.08	0.30	0.8
*MMP12*	Matrix metallopeptidase 12	-	0.79	0.84	0.9
*MMP13*	Matrix metallopeptidase 13	-	0.03	0.14	2.6
*MMP15*	Matrix metallopeptidase 15	-	0.15	0.35	0.5
*MMP16*	Matrix metallopeptidase 16	-	0.57	0.67	0.6
*MMP17*	Matrix metallopeptidase 17	-	0.56	0.67	0.8
*MMP20*	Matrix metallopeptidase 20	-	0.53	0.67	0.7
*MMP21*	Matrix metallopeptidase 21	-	0.13	0.33	1.5
*MMP23A/B*	Matrix metallopeptidase 23A/B	-	0.34	0.49	0.8
*MMP24*	Matrix metallopeptidase 2	-	0.29	0.49	0.6
*MMP25*	Matrix metallopeptidase 25	-	0.18	0.40	0.7
*MMP26*	Matrix metallopeptidase 26	-	0.34	0.49	0.8
*MMP27*	Matrix metallopeptidase 27	-	0.33	0.49	0.8

**Table 7:** Change in collagen gene expression in human tendinopathy

Gene Symbol	Description		p-value	q-value	Fold Change
Regulated					
*COL1A1*	Collagen, type I, alpha 1	Expressed	9.37E-03	3.96E-02	1.7
*COL1A2*	Collagen, type I, alpha 2	Expressed	4.45E-03	3.16E-02	1.6
*COL3A1*	Collagen, type III, alpha 1	Expressed	1.50E-02	4.28E-02	2.0
*COL4A1*	Collagen, type IV, alpha 1	Expressed	1.91E-02	4.53E-02	2.7
*COL4A2*	Collagen, type IV, alpha 2	Expressed	6.09E-03	3.16E-02	2.3
*COL5A1*	Collagen, type V, alpha 1	Expressed	1.20E-02	4.28E-02	2.0
*COL5A2*	Collagen, type V, alpha 2	Expressed	3.99E-03	3.16E-02	2.1
*COL5A3*	Collagen, type V, alpha 3	Expressed	3.02E-03	3.16E-02	2.0
*COL6A1*	Collagen, type VI, alpha 1	Expressed	2.93E-02	6.18E-02	1.8
*COL6A2*	Collagen, type VI, alpha 2	Expressed	3.62E-02	6.89E-02	1.8
*COL6A3*	Collagen, type VI, alpha 3	Expressed	5.45E-03	3.16E-02	1.7
*COL8A1*	Collagen, type VIII, alpha 1	Expressed	2.49E-02	5.57E-02	2.0
*COL8A2*	Collagen, type VIII, alpha 2	Expressed	1.48E-02	4.28E-02	1.8
*COL10A1*	Collagen, type X, alpha 1	Expressed	1.69E-02	4.28E-02	2.1
*COL12A1*	Collagen, type XII, alpha 1	Expressed	4.10E-03	3.16E-02	2.1
*COL18A1*	Collagen, type XVIII, alpha 1	Expressed	6.65E-03	3.16E-02	2.2
*COL27A1*	Collagen, type XXVII, alpha 1	Expressed	1.20E-03	3.16E-02	2.5
Not Regulated					
*COL7A1*	Collagen, type VII, alpha 1	Expressed	0.96	0.96	1.0
*COL11A1*	Collagen, type XI, alpha 1	Expressed	0.58	0.71	1.2
*COL15A1*	Collagen, type XV, alpha 1	Expressed	0.51	0.64	1.2
*COL16A1*	Collagen, type XVI, alpha 1	Expressed	0.12	0.19	1.4
*COL21A1*	Collagen, type XXI, alpha 1	Expressed	0.34	0.45	1.4
Expressed below limited of detection					
*COL2A1*	Collagen, type II, alpha 1	-	0.21	0.31	0.5
*COL4A3*	Collagen, type IV, alpha 3	-	0.15	0.23	0.6
*COL4A4*	Collagen, type IV, alpha 4	-	0.90	0.96	1.0
*COL4A5*	Collagen, type IV, alpha 5	-	0.71	0.84	0.9
*COL4A6*	Collagen, type IV, alpha 6	-	0.94	0.96	1.0
*COL9A1*	Collagen, type IX, alpha 1	-	0.94	0.96	1.0
*COL9A2*	Collagen, type IX, alpha 2	-	0.05	0.08	1.9
*COL11A2*	Collagen, type XI, alpha 2	-	0.12	0.19	0.7
*COL13A1*	Collagen, type XIII, alpha 1	-	0.08	0.13	2.0
*COL14A1*	Collagen, type XIV, alpha 1	-	0.02	0.04	1.8
*COL17A1*	Collagen, type XVII, alpha 1	-	0.33	0.45	1.3
*COL19A1*	Collagen, type XIX, alpha 1	-	0.02	0.04	0.5
*COL22A1*	Collagen, type XXII, alpha 1	-	0.03	0.07	0.5
*COL23A1*	Collagen, type XXIII, alpha 1	-	0.75	0.84	1.0
*COL24A1*	Collagen, type XXIV, alpha 1	-	0.33	0.45	1.3
*COL25A1*	Collagen, type XXV, alpha 1	-	0.76	0.84	0.9

Diseased tendons showed increased expression of a number of relevant and important signaling pathways; for example the fibroblast growth factors *FGFR1 *(1.7-fold) and *FGFR2 *(2.7-fold), and genes in the Notch signaling pathway, including *JAG1 *(3.1-fold) and *Notch3 *(3.6-fold). There was also evidence for increased WNT signaling. Among the highest regulated transcripts, four are involved in WNT signaling: *WISP1 *(4.2-fold), *DKK3 *(3.6-fold), *WNT3 *(3.6-fold), and *LEF1 *(4.1-fold). Additionally, tendinopathy tissue showed differential expression of other WNT pathway genes, including increased expression of *CTNNB *(1.5-fold), *WNT5a *(2.7-fold), and *FZD1 *(1.7-fold) and decreased expression of *LRP5 *(0.59-fold) and *FRZB *(0.35-fold).

We found little evidence for endochondral ossification even though this has been reported in Achilles and patella tendinopathy [21]. Histological evaluation failed to identify any gross areas of ossification in any of the patients and RNA analysis did not show increased expression of the bone markers osteocalcin or osterix. However, expression of *CBFA1*, a transcription factor associated with osteoblast differentiation, was increased 1.8-fold and that of *RANKL*, a key factor for osteoclast differentiation and activation, was increased 4.0-fold in diseased tendons.

In addition to the global pathway analysis, we specifically investigated the regulation of kinases in diseased tendons (Table 8). The most statistically significant regulated kinase is diseased tendons is the thousand and one amino acid kinase (*TAOK1*), which showed a 1.7-fold increase in expression. TAOK1 activates the p38 MAP kinase pathway and tendonopathy tissue also showed increased expression of multiple MAP kinases, including *MAP3K2 *(1.8-fold), *MAP4K5 *(1.9-fold), and *MAPK8 *(2.0-fold).

**Table 8:** Kinases regulated in human tendinopathy

Gene Symbol	Description	pValue	q-value	Fold Change
*ABL2*	v-abl Abelson leukemia viral oncogene homolog 2	4.79E-03	7.83E-03	31.0
*BMPR2*	Bone morphogenetic protein receptor, type II	4.10E-03	7.83E-03	27.0
*CAV2*	Caveolin 2	1.22E-02	1.44E-02	43.0
*CDK6*	Cyclin-dependent kinase 6	1.37E-02	1.53E-02	24.0
*CSNK1D*	Casein kinase 1, delta	5.21E-03	8.13E-03	19.0
*CSNK1G1*	Casein kinase 1, gamma 1	8.18E-03	1.03E-02	40.0
*DCLK1*	Doublecortin-like kinase 1	3.78E-03	7.83E-03	28.0
*EML4*	Echinoderm microtubule associated protein like 4	9.69E-04	3.15E-03	41.0
*EPS8*	Epidermal growth factor receptor substrate 8	1.96E-04	1.67E-03	9.0
*FGFR1*	Fibroblast growth factor receptor 1	5.64E-03	8.46E-03	1.7
*ILK*	Integrin-linked kinase	1.00E-04	1.67E-03	1.6
*JAK3*	Janus kinase 3	4.62E-03	7.83E-03	1.8
*KIAA0220*	PI-3-kinase-related kinase SMG-1	2.70E-03	7.02E-03	1.6
*KIAA0999*	KIAA0999 protein	1.62E-03	4.86E-03	2.1
*MAP3K2*	Mitogen-activated protein kinase kinase kinase 2	4.82E-03	7.83E-03	1.8
*MAP4K5*	Mitogen-activated protein kinase kinase kinase 5	1.31E-02	1.50E-02	1.9
*MAPK8*	Mitogen-activated protein kinase 8	1.42E-02	1.54E-02	2.0
*NAGK*	N-acetylglucosamine kinase	4.16E-03	7.83E-03	1.8
*PALM2-AKAP2*	A kinase (PRKA) anchor protein 2	2.31E-02	2.37E-02	1.9
*PANK2*	Pantothenate kinase 2	6.39E-04	2.67E-03	1.6
*PAPSS1*	3'-phosphoadenosine 5'-phosphosulfate synthase 1	2.35E-04	1.67E-03	1.8
*PDLIM5*	PDZ and LIM domain 5	8.94E-03	1.09E-02	1.9
*PFTK1*	PFTAIRE protein kinase 1	4.74E-03	7.83E-03	1.6
*PGAM1*	Phosphoglycerate mutase 1 (brain)	4.81E-04	2.35E-03	1.6
*PI4K2B*	Phosphatidylinositol 4-kinase type 2 beta	7.80E-03	1.02E-02	1.7
*PIK3C2A*	Phosphoinositide-3-kinase, class 2, alpha	2.28E-02	2.37E-02	1.5
*PIP4K2A*	Phosphatidylinositol-5-phosphate 4-kinase, Iia	7.52E-04	2.67E-03	1.5
*PRKAA1*	Protein kinase, AMP-activated, alpha 1	5.32E-02	5.32E-02	1.7
*PRKD1*	Protein kinase D1	7.86E-03	1.02E-02	1.8
*PTK9*	Twinfilin, actin-binding protein, homolog 1	1.62E-04	1.67E-03	1.7
*RIPK2*	Receptor-interacting serine-threonine kinase 2	1.84E-03	5.11E-03	1.6
*RIPK5*	Receptor interacting protein kinase 5	5.91E-03	8.53E-03	1.7
*SGMS2*	Sphingomyelin synthase 2	3.38E-03	7.76E-03	2.3
*SHC1*	Src homology 2 domain containing protein 1	2.99E-04	1.67E-03	1.7
*SOCS3*	Suppressor of cytokine signaling 3	3.07E-03	7.48E-03	1.7
*SSA2*	TROVE domain family, member 2	2.72E-04	1.67E-03	1.6
*TAOK1*	TAO kinase 1	1.51E-04	1.67E-03	1.7
*TRIM27*	Tripartite motif-containing 27	6.94E-04	2.67E-03	1.6
*TTN*	Titin	6.67E-03	9.29E-03	0.2

## Discussion

We have evaluated gene expression and histological changes associated with human tendinopathy. While gene expression changes in human tendinopathy have previously been described [13,22,23], this study represents the largest and most complete study to date. However, our study has several limitations. First, control tendons were taken from the same joints as the diseased tendons. These control tendons appeared normal macroscopically but their proximity to the diseased tendon may have some effect on the control tendon. Furthermore, the control tendon was an anatomically distinct tendon exposed to different loads and strains and it is not known how this may affect the gene expression profile. Moreover, the tendon specimens were collected from a heterogeneous population with differences in age, gender, symptoms, duration of disease, and physical activity (Table 1). While changes in gene expression were not related to any of these differences, they all contribute to variations in the data. Given the vast variation in the human population, we reasoned that control samples from the same patients would be a better control for interpatient variability than tendons from control patients without any tendon symptoms.

Progression of tendinopathy is dependent on extracellular matrix integrity and remodeling of the tendon is a common feature of tendinopathy [24]. The extracellular matrix of normal tendons consists of many of structural proteins (collagens) and proteoglycans. Collagen is the main component of tendons and type I collagens account for 65% to 80% of the total tendon mass [25]. mRNA levels for type I and type III collagens (*COL1A1, COL1A2*, and *COL3A1*) were increased in the diseased tendons. Tendons also contain several proteoglycans, although none of the proteoglycans analyzed (including aggrecan, versican, decorin, biglycan, fibromodulin, and lumican) showed increased expression in diseased tendons. In contrast, expression of the non-collagen glycoproteins fibronectin, tenascin C, fibrillin, and laminin was increased in diseased tendons. Consistent with active remodeling, increased MMP activity, particularly of MMP2 and MMP9, has been associated with ruptured Achilles tendon [26]. Diseased tendons showed increased expression of several matrix associated proteins including *MMP19, MMP9, MMP13, MMP14*, and *MMP2*. In contrast, *MMP3 *expression was decreased in tendinopathy in both our study and in others [13,22].

We can begin to speculate how MMP activity is regulated in tendinopathy tissue. Diseased tissues showed increased expression of components of the JAK/STAT pathway (*STAT3*, 1.9-fold, and *JAK3*, 1.8-fold). The JAK/STAT pathway is known to activate *IL4R *[27], which induces expression of *IL13RA2 *[28]. Expression of both *IL4R *and *IL13RA2 *was elevated in diseased tissue (3.2- and 3.5-fold, respectively). IL13, secreted by T helper type 2 cells, has anti-inflammatory properties and through interaction with its receptor IL13RA2 induces certain MMPs [29] including *MMP9 *and *MMP14 *[30]. Together, these findings suggest that activation of the JAK/STAT pathway may lead to increased MMP activation.

Chard et al. previously suggested that tendon pathology is linked to formation of excessive fibrocartilage [31]; however, other prior studies [22] and our current study found little evidence of chondrocyte-like cells. Tendinopathy tissue showed decreased expression of a number of cartilage markers including *COMP*, but expression of other cartilage markers such as aggrecan was not altered. In contrast, expression of type I collagen was increased, further supporting the absence of increased chondrocytic cells in diseased tendons. This is in contrast to an overuse animal model of tendinopathy [32,33] that suggested increased formation of fibrocartilage associated with overuse. This discrepancy can possibly be explained by differences in disease severity. The human samples were collected during surgery following tendon tear or rupture and represent late stage disease in which surgical intervention became necessary, while the animal models represent earlier development of disease. Furthermore, the human tendon samples represent small biopsies, mostly from the midsubstance. One might expect any increase in fibrocartilage to occur near the bone insertion site, an area from which samples were not collected.

Global pathway analysis identified novel pathways that are differentially regulated in human tendinopathy including regulation of genes involved in WNT signaling. Tendinopathy is characterized by increased cellular infiltration and proliferation. Increases in WNT signaling can promote proliferation and maintain cells in an undifferentiated state [34] and the regulation of multiple WNT signaling genes suggests that this pathway is activated in tendinopathy. Pathway analysis also suggested an increased regulation of genes involved in integrin signaling. Integrins are receptors involved in cell adhesion to the extracellular matrix and transmit extracellular signals into the cell to regulate gene expression. While many of the genes encoding integrins were only slightly upregulated, expression of integrin beta 1 (fibronectin receptor) was significantly increased in tendinopathy tissue.

We found little direct evidence to support an inflammatory response in established human tendinopathy. Histological evaluation did not identify a significant number of inflammatory cells and analysis of the gene expression of inflammatory cytokines identified only a handful of cytokine genes that were differentially regulated in diseased tendons, although a number of these are implicated in tendon function and tendon healing. Expression of the proinflammatory cytokine *IL17D *was reduced in diseased tendons. IL17 increases the turnover of type I collagen through both inhibiting its synthesis and promoting its breakdown [35], and members of the IL17 cytokine family members are inhibitors of human hematopoietic progenitor proliferation [36]. The oncostatin M receptor was significantly upregulated in diseased tendons. Oncostatin M contributes to the release of proteoglycans and the breakdown of collagens [37]. IL6 is a pro-inflammatory cytokine important for tendon healing; lack of IL6 prevents proper tendon healing [38]. Evidence for aberrant regulation of the IL6 pathway in damaged tendons includes decreased expression of the *IL6 *receptor. *IL6 *can also signal through *STAT3*, which was upregulated in diseased tendons. *STAT3 *expression has also been identified in ruptured rotator cuff. However, activation of STAT3 is mainly induced by proliferating vessels [39], and since diseased tendons have increased vasculature, many of the observed changes in cytokine expression may simply be due to this change in vasculature. Therefore, whether these cytokines play a direct role in tendonopathy requires further study. In addition to the lack of evidence for direct regulation of many pro-inflammatory cytokines, there is also indirect evidence for the absence of inflammatory cytokine activity. The lack of expression of *MMP1 *and *MMP13*, which are known to be induced by inflammatory cytokines [40], further supports the proposal that pro-inflammatory cytokines do not play a major role in tendinopathy at this late stage of disease.

## Conclusions

In this paper we describe the global transcriptome of human tendinopathy. Although we have identified a number of genes that are differentially regulated, the ultimate roles of these genes and pathways in tendon disease are yet to be determined. However, we have provided a resource that we and other investigators can use to explore the molecular changes associated with tendinopathy.

## Competing interests

Howard Seeherman, Jian Li, Joanne Archambault and Scott Jelinsky were employed by Wyeth Research/Pfizer at the time of this research. Scott A Rodeo received research funding from Wyeth at the time of this study.

## Authors' contributions

HJS, JMA and SAR worked on the study design. SAR recruited patients and collected the samples. LVG wrote the IRB and informed consents and help collect the samples. JL performed the histology analysis. SAJ performed the microarray analysis and drafted the manuscript. All authors have read and approved the final manuscript.

## Pre-publication history

The pre-publication history for this paper can be accessed here:

http://www.biomedcentral.com/1471-2474/12/86/prepub
